# Digital Innovations in Nursing Workforce Management: A Scoping Review of Advanced Applications and Implementation Challenges

**DOI:** 10.1155/jonm/1858294

**Published:** 2026-09-14

**Authors:** Rafat Rezapour-Nasrabad, Sina Nasrollahi-Nasrabad

**Affiliations:** ^1^ Department of Psychiatric Nursing and Management, School of Nursing and Midwifery, Shahid Beheshti University of Medical Sciences, Tehran, Iran, sbmu.ac.ir; ^2^ Department of Medicine and Surgery, University of Parma, Via Volturno 39, Parma 43125, Italy, unipr.it

**Keywords:** artificial intelligence, human resource management, nurse staffing, nursing workforce management, scoping review, workforce planning

## Abstract

**Objective:**

Artificial intelligence (AI) is increasingly being adopted to support healthcare workforce planning and operational decision‐making. However, evidence regarding its application specifically within nursing workforce management remains fragmented across diverse disciplines and publication types. This scoping review aimed to map the current evidence on AI applications in nursing workforce management, identify major application domains, synthesise reported organisational outcomes and implementation challenges and highlight priorities for future research.

**Design:**

Scoping review.

**Methods:**

The review was conducted in accordance with the Arksey and O’Malley methodological framework, subsequent methodological enhancements by Levac and colleagues, the Joanna Briggs Institute guidance and the PRISMA Extension for Scoping Reviews (PRISMA‐ScR). A comprehensive literature search was undertaken in PubMed, Scopus, Web of Science, CINAHL and IEEE Xplore from database inception to May 2025, with a supplementary top‐up search executed in August 2026 to ensure currency. Two reviewers independently screened studies, extracted data using a standardised data‐charting form and synthesised findings through descriptive numerical analysis and thematic synthesis.

**Results:**

Twenty‐eight studies met the eligibility criteria. AI applications were identified across six principal workforce management domains: workforce planning and demand forecasting, nurse scheduling and rostering, workload optimisation, burnout and workforce sustainability, workforce analytics and managerial decision support. Most publications described conceptual models, pilot projects, qualitative investigations or review‐based evidence, whereas relatively few evaluated implemented AI systems in routine nursing management practice. Commonly reported organisational benefits included improvements in workforce allocation, scheduling transparency, operational efficiency and data‐informed managerial decision‐making. Frequently reported implementation challenges included ethical concerns, organisational readiness, data quality, interoperability, digital competence and user acceptance.

**Conclusion:**

Current evidence suggests that AI has considerable potential to support nursing workforce management; however, empirical evidence regarding implementation effectiveness remains limited. Future research should prioritise prospective implementation studies, rigorous evaluation of workforce outcomes and development of transparent, nurse‐centred AI governance frameworks.

**Implications for Nursing Management:**

AI‐enabled workforce management may assist nurse leaders in efforts to improve staffing decisions, potentially enhancing operational efficiency and supporting efforts toward more equitable workload distribution. Successful implementation requires organisational readiness, robust governance, multidisciplinary collaboration and meaningful involvement of nurses throughout system design and evaluation.

## 1. Introduction

Healthcare systems worldwide continue to face unprecedented workforce pressures arising from persistent nursing shortages, increasing patient complexity, population ageing and growing demands for high‐quality care. These challenges have intensified the need for innovative workforce management strategies that support timely, evidence‐informed managerial decision‐making. Nursing workforce management encompasses a broad range of organisational activities, including workforce planning, staffing, scheduling, workload allocation, recruitment, retention, performance management and workforce sustainability. The effectiveness of these processes directly influences patient safety, quality of care, nurse well‐being, organisational efficiency and financial performance [1, 2]. Artificial intelligence (AI) has emerged as one of the most influential technologies driving digital transformation across health care. Rather than replacing professional judgement, contemporary AI systems increasingly serve as decision‐support tools that can analyse large, complex datasets, identify hidden patterns, generate predictions and optimise operational processes. Recent advances in machine learning, predictive analytics, optimisation algorithms, natural language processing and intelligent decision‐support systems have created new opportunities to improve workforce planning, anticipate staffing demands, optimise scheduling and support strategic nursing management [3–5].

Although AI has been extensively investigated in clinical decision support, medical imaging, disease prediction and patient monitoring, its application within nursing workforce management has received comparatively less attention. Existing studies suggest that AI may support workforce forecasting, dynamic staffing allocation, workload optimisation, burnout prediction and scheduling transparency. AI‐driven workforce analytics may also assist nurse managers in responding proactively to fluctuations in patient demand, workforce availability and organisational priorities [5–7]. Nevertheless, many reported applications remain at the conceptual, simulation or pilot stage, and evidence regarding large‐scale implementation within healthcare organisations remains limited.

Several recent reviews have examined AI in nursing from broader perspectives, including clinical practice, nursing education, digital transformation and professional competencies [7, 8]. More recently, Kiwanuka et al. [9] explored leadership and AI integration within nursing workforce management. However, existing reviews have generally focused on specific aspects of AI adoption or have combined workforce management with broader digital health topics. Consequently, the literature remains fragmented regarding the range of AI technologies employed, the workforce management domains addressed, the maturity of implementation and the organisational challenges associated with adoption. Furthermore, limited synthesis has been undertaken to distinguish conceptual developments from empirical implementation studies or to identify priorities for future nursing management research.

Given the accelerating digital transformation of healthcare organisations and increasing expectations placed upon nurse managers to make data‐informed workforce decisions, a comprehensive mapping of current evidence is both timely and necessary. Understanding where AI has been successfully applied, where evidence remains limited and what barriers impede implementation is essential for guiding future organisational investment, research and policy development.

Accordingly, this scoping review aimed to systematically map the existing literature on AI applications in nursing workforce management, synthesise current evidence across major workforce management domains, identify reported organisational outcomes and implementation challenges and highlight evidence gaps requiring future investigation. By integrating evidence across diverse study designs and implementation contexts, this review provides nurse managers, healthcare leaders, researchers and policymakers with a comprehensive overview of the current state of AI‐enabled nursing workforce management and future directions for evidence‐informed digital transformation.

## 2. Aim and Research Questions

### 2.1. Aim

This scoping review aimed to systematically map and synthesise the existing evidence regarding the application of AI in nursing workforce management. In particular, the review sought to identify the principal application domains of AI, describe the technologies employed, examine reported organisational outcomes and implementation challenges and identify gaps that should inform future nursing management research, policy and practice.

### 2.2. Research Questions

The following research questions guided this review:

1. Which AI technologies have been applied within nursing workforce management?
2. Which domains of nursing workforce management (e.g., workforce planning, staffing, scheduling, workload management, recruitment, retention and decision support) have been addressed using AI?
3. What organisational, managerial and workforce outcomes have been reported following AI implementation?
4. What ethical, organisational, technological and implementation challenges have been identified during AI adoption in nursing workforce management?
5. What evidence gaps and future research priorities emerge from the current literature?

## 3. Methods

### 3.1. Study Design

This scoping review was conducted in accordance with the methodological framework proposed by Arksey and O’Malley [10], incorporating subsequent methodological refinements described by Levac and colleagues, as well as the recommendations of the Joanna Briggs Institute (JBI) Manual for Evidence Synthesis. Reporting followed the Preferred Reporting Items for Systematic Reviews and Meta‐Analyses Extension for Scoping Reviews (PRISMA‐ScR).

A scoping review methodology was considered appropriate because the objective of this study was to comprehensively map the breadth, characteristics and nature of the available evidence regarding AI applications in nursing workforce management rather than to evaluate intervention effectiveness or estimate pooled effects.

A review protocol was developed before commencement of the review to guide study identification, eligibility assessment, data charting and evidence synthesis. Although the review protocol was not prospectively registered, the a priori protocol detailing the review objectives, eligibility criteria and analytical methods is provided as Supporting File 3 to ensure full transparency regarding the prespecified methodology.

### 3.2. Eligibility Criteria

Eligibility criteria were developed using the Population–Concept–Context (PCC) framework recommended by the JBI for scoping reviews.

### 3.3. Population

Studies involving registered nurses, nurse managers, nursing teams, nursing departments or healthcare organisations in which nursing workforce management constituted the primary focus were eligible.

### 3.4. Concept

AI was broadly defined as computational systems capable of performing tasks that normally require human cognitive processes, including machine learning, deep learning, predictive analytics, optimisation algorithms, natural language processing, intelligent decision‐support systems, reinforcement learning and related AI‐enabled analytical techniques.

Studies describing conventional digital technologies, electronic health records, workforce information systems or scheduling software without explicit AI functionality were excluded.

Nursing workforce management was defined as organisational activities related to workforce planning, staffing, scheduling, workload management, recruitment, retention, workforce analytics, performance management, resource allocation and managerial decision‐making.

### 3.5. Context

Studies conducted in hospitals, community healthcare organisations, long‐term care facilities, academic health centres and other healthcare settings were eligible.

### 3.6. Operational Definitions

For this review, *AI* was defined as computational systems capable of performing tasks that typically require human cognitive processes, including learning from data, pattern recognition, prediction, optimisation, natural language processing or decision support [3, 4, 7, 8]. Eligible AI technologies included machine learning algorithms, deep learning models, predictive analytics, optimisation algorithms, intelligent decision‐support systems and other computational approaches demonstrating adaptive or algorithm‐driven reasoning beyond conventional rule‐based programming [3, 5, 11, 12].

Nursing workforce management was defined as organisational activities related to planning, staffing, scheduling, workload allocation, workforce forecasting, recruitment, retention, performance management and managerial decision‐making concerning the nursing workforce [5, 9]. Studies were eligible only if AI technologies were applied directly to one or more of these workforce management functions.

Studies describing conventional digital health technologies, electronic health records, hospital information systems or workforce software without an identifiable AI component were excluded. In particular, systems based solely on fixed rule‐based logic, deterministic automation or standard digital record management without machine learning, predictive modelling, optimisation algorithms or other AI‐driven computational methods were not considered eligible for inclusion [11, 13]. *Furthermore, although the search strategy intentionally included the broad term ‘algorithm’ to capture early predictive workforce modelling, studies relying solely on fixed, deterministic mathematical rules without adaptive or machine-learning capabilities were excluded during full-text screening.* Borderline studies utilising algorithm‐driven predictive modelling or workflow recognition were retained only if the original publication explicitly described the computational logic as extending beyond conventional manual or rule‐based calculations. In cases where the classification of a technology as AI was unclear, the full text was independently assessed by two reviewers, and eligibility was determined by consensus based on the operational definition adopted for this review.

### 3.7. Types of Evidence Sources

To comprehensively map the emerging evidence base, empirical quantitative studies, qualitative studies, mixed‐methods studies, implementation studies, modelling studies, simulation studies and evidence syntheses (systematic reviews, scoping reviews and integrative reviews), *as well as policy analyses and position papers,* were eligible.

Editorials, opinion papers, conference abstracts, letters to editors, protocols, dissertations and studies without full‐text availability were excluded.

Methodological guidance papers (e.g., PRISMA‐ScR [14], Arksey & O’Malley, Levac et al. [15], JBI Manual [16]) were used exclusively to inform the review methodology and were not included in the evidence synthesis.

### 3.8. Information Sources and Search Strategy

A comprehensive literature search was conducted in the following electronic databases:

• PubMed/MEDLINE
• Scopus
• Web of Science Core Collection
• CINAHL Complete
• IEEE Xplore

Search strategies were developed collaboratively with a research librarian following an iterative process. Controlled vocabulary (e.g., Medical Subject Headings [MeSH] in PubMed and CINAHL Subject Headings) was combined with free‐text keywords using Boolean operators (AND, OR) to maximise sensitivity. The search strategy combined three major concepts: (1) AI, (2) nursing and (3) workforce management. Given the rapid emergence of specific AI subfields, the search strategy intentionally utilised high‐level, overarching terms (e.g., ‘artificial intelligence’, ‘machine learning’) to comprehensively capture all subdisciplines, including generative AI and large language models (LLMs). Specific proprietary terms (e.g., ‘ChatGPT’) were deliberately excluded from the search strings to avoid an excess of nonempirical literature (e.g., opinion pieces), as rigorous studies applying these emerging technologies to workforce management inherently index under the broader ‘artificial intelligence’ descriptors. *No start-date restriction was applied, meaning the search spanned from the inception of each database. The final search was executed on 31 May 2025*.

Following the initial search, a top‐up search was conducted from 1 June 2025 to 10 August 2026 to ensure the evidence base reflects the latest developments in this rapidly evolving field. No additional new studies were identified in the updated search for inclusion in the analysis.

The complete database‐specific search strategies, including all search strings, syntax, filters applied and the number of results retrieved from each database, are provided in Supporting File 1.

Reference lists of included studies were also manually screened to identify additional relevant publications.

### 3.9. Study Selection

All retrieved citations were exported into EndNote (Clarivate Analytics), where duplicate records were identified and removed.

Study selection was undertaken in two sequential stages by two reviewers working independently.

During the first stage, titles and abstracts were screened against the predefined eligibility criteria.

Potentially relevant studies progressed to full‐text assessment. During the second stage, full texts were independently reviewed by the same reviewers to determine final eligibility.

Disagreements during either stage were resolved through discussion and consensus. When consensus could not initially be reached, the study was re‐evaluated jointly until agreement was achieved.

Reasons for exclusion at the full‐text stage were documented and are presented in the PRISMA‐ScR flow diagram.

### 3.10. Data Charting

Data extraction was performed independently by two reviewers using a standardised data‐charting form specifically developed for this review.

Before formal extraction commenced, the charting form was pilot‐tested using five randomly selected studies to ensure consistency, completeness and clarity. Minor modifications were subsequently made to improve data capture.

The following information was extracted:

• Author(s)
• Publication year
• Country
• Study design
• Evidence type
• Healthcare setting
• AI technology
• Workforce management domain
• AI maturity level
• Key findings

To ensure consistent and replicable classification, the ‘AI maturity level’ has been operationally defined using the following criteria:

• *Conceptual*: Studies describing theoretical frameworks, proposed models or policy analyses regarding AI in nursing workforce management, without empirical data from an active AI system.
• *Pilot*: Studies that test an AI system in a controlled, simulated or small‐scale environment primarily to assess feasibility, usability or user acceptance before routine deployment.
• *Implemented*: Studies where the AI system is deployed in routine practice, and the research focuses on the integration process, user experiences or descriptive outcomes (e.g., cross‐sectional satisfaction surveys) rather than employing a rigorous comparative or longitudinal design to measure system performance.
• *Evaluated*: Studies in which the AI system is in routine practice, and the research employs specific methodological designs (e.g., time‐motion studies, pre–post‐comparisons, accuracy testing against gold standards) to objectively measure the technical performance or operational impact of the AI system.

It is important to distinguish between evidence type and AI maturity. A study may employ an empirical design (e.g., a qualitative study exploring nurse perceptions), whereas the AI component itself remains at the conceptual maturity stage because no active AI system was evaluated. Therefore, studies classified under ‘conceptual’ maturity are not opinion papers, but rather primary empirical investigations focused on the conceptualisation, readiness or organisational context of AI before its deployment.

Following independent extraction, the reviewers compared completed forms and resolved discrepancies through discussion until consensus was achieved.

### 3.11. Data Synthesis

Extracted data were synthesised using descriptive numerical analysis and thematic synthesis.

Descriptive analysis summarised

• publication year
• country of origin
• study design
• evidence type
• AI technologies
• workforce management domains

Thematic synthesis was subsequently undertaken to identify recurring application areas, implementation challenges, organisational outcomes, ethical considerations and research gaps.

Rather than evaluating intervention effectiveness, the synthesis focused on mapping the characteristics and distribution of the available evidence.

Consistent with scoping review methodology, no meta‐analysis was undertaken.

To avoid double counting of evidence, primary studies and secondary evidence sources were analysed separately. Primary studies were used exclusively for the numerical descriptive analysis and the AI maturity cross‐tabulation (Table 1). The 14 secondary evidence sources were utilised strictly for contextual and conceptual mapping such as identifying broader ethical frameworks, historical trends and policy landscapes rather than being treated as independent observations of AI implementation outcomes.

**TABLE 1:** Cross‐tabulation of AI maturity levels across workforce management domains (primary studies, *n* = 14).

Workforce management domain	Conceptual	Pilot	Implemented	Evaluated	Total
Workforce planning and demand forecasting	0	0	1	0	1
Nurse scheduling and rostering	1	2	1	0	4
Workload management	0	0	0	2	2
Workforce sustainability and burnout prevention	0	0	0	0	0
Workforce analytics	1	0	0	0	1
Managerial decision support	3	1	2	0	6
Total	5	3	4	2	14

*Note:* Numbers represent the count of primary studies. Secondary evidence sources (*n* = 14) were excluded from this matrix as their maturity level is not applicable.

### 3.12. Quality Appraisal

Consistent with the objectives of a scoping review and current PRISMA‐ScR and JBI recommendations, no formal methodological quality appraisal or risk‐of‐bias assessment was performed.

The purpose of this review was to map the breadth and characteristics of the available evidence rather than determine the effectiveness of interventions or generate practice recommendations.

Accordingly, findings should be interpreted as an overview of the existing evidence landscape rather than as evidence of comparative effectiveness.

## 4. Results

### 4.1. Study Selection

The database search identified 620 records. Following removal of duplicate records, 452 unique citations underwent title and abstract screening. Of these, 360 records were excluded because they did not meet the eligibility criteria. The remaining 92 articles were assessed in full text.

Following full‐text review, 64 studies were excluded for reasons including lack of explicit AI application, focus on digital technologies without AI functionality, non–healthcare settings, conference abstracts, editorials, protocols and articles not addressing nursing workforce management. Ultimately, 28 publications were included in the final evidence synthesis.

**FIGURE 1:**
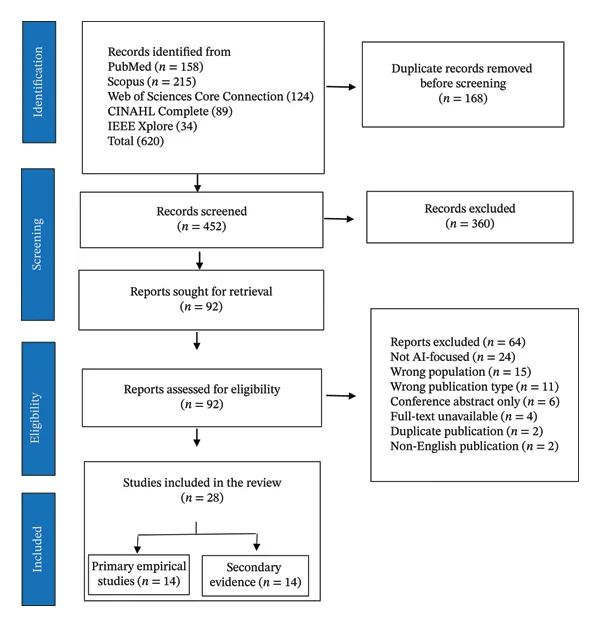
PRISMA 2020 flow diagram of study selection. Adapted from Page MJ, McKenzie JE, Bossuyt PM, et al. The PRISMA 2020 statement: an updated guideline for reporting systematic reviews. BMJ 2021; 372: n71.doi:10.1136/bmj. n71. Note: A supporting top‐up search executed on 10 August 2026 identified no new records after deduplication; therefore, no additional records required screening, and the PRISMA‐ScR flow diagram reflects only the original search.

The study selection process is presented in the PRISMA‐ScR flow diagram (Figure 1). *Consequently, the top-up search yielded zero new records, and the PRISMA-ScR flow diagram* (Figure 1) *exclusively reflects the yield from the original search.*

### 4.2. Characteristics of Included Studies

The 28 included publications were published between 2010 and 2025, with a marked increase in publication frequency after 2022, reflecting growing international interest in AI‐supported nursing workforce management.

The included literature represented a broad range of evidence types, including qualitative studies, mixed‐methods studies, cross‐sectional surveys, implementation studies, observational research, literature reviews, scoping reviews, integrative reviews, mapping reviews, policy analyses and position papers.

Most publications originated from high‐income countries, particularly the United States, the United Kingdom, Canada, Taiwan and New Zealand. However, notably, the evidence base also includes representation from Middle Eastern and North African (MENA) countries, including Saudi Arabia, Egypt, Qatar, Jordan and Lebanon. Although this MENA representation provides valuable insights into AI adoption in these contexts, a broader geographical imbalance persists, as evidence from low‐ and middle‐income countries outside of the MENA region remains entirely absent.

Tables 2 and 3 summarise the characteristics of all included studies.

**TABLE 2:** Characteristics of primary studies on artificial intelligence in nursing workforce management.

No.	Author (year)	Country	Study design	Evidence type	Healthcare setting	AI technology/AI component	Workforce management domain	AI maturity level	Key findings
1	Aksu and Dinç (2025) [17]	Türkiye	Design‐based study with pilot evaluation	Primary Study	Hospital	AI‐assisted mobile scheduling application	Nurse scheduling	Pilot	Developed and piloted an AI‐assisted scheduling application that improved fairness, transparency, user satisfaction and reduced roster preparation time
2	Göktepe and Sarıköse (2025) [6]	Canada	Qualitative study	Primary study	Hospital	AI‐enabled workforce analytics	Workforce management	Conceptual	Trust, explainability and organisational readiness strongly influenced AI acceptance among nurse managers
3	Hunstein and Fiebig (2024) [18]	Germany	Multicentre observational study	Primary study	Hospitals	Machine learning	Workload prediction	Evaluated	Machine learning algorithms accurately predicted nursing workload and staffing demand
4	Spurlock et al. (2022) [19]	United States	Qualitative thematic analysis	Primary study	National (multisetting)	Artificial intelligence (general AI applications in nursing)	Workforce readiness and operational management	Conceptual	Identified major themes regarding nurses’ perceptions of AI, highlighting the need for nurse involvement in AI design, concerns about workforce displacement and the necessity of AI competency for future nursing leadership and management
5	Kang et al. (2025) [20]	South Korea	Cross‐sectional evaluation	Primary study	Hospital	AI scheduling platform	Shift scheduling	Implemented	AI‐assisted scheduling increased nurse satisfaction and scheduling efficiency
6	Ramadan et al. (2024) [21]	Saudi Arabia	Qualitative study	Primary study	Hospital	Artificial intelligence (Technology Acceptance Model for AI in Nursing [TAM‐AIN])	Nursing leadership, organisational readiness and workforce management	Conceptual	Explored registered nurses’ perceptions of AI adoption and identified organisational readiness, leadership support, digital competence, infrastructure, ethical governance and workforce training as critical enablers. The study proposed the Technology Acceptance Model for AI in Nursing (TAM‐AIN) to facilitate successful organisational AI implementation
7	Tu et al. (2025) [22]	Taiwan	Implementation/evaluation study	Primary study	Hospital	Generative AI tools	Nursing workflow and workload efficiency	Implemented	GenAI‐assisted nursing handover substantially reduced documentation time, improved work efficiency and demonstrated practical scalability for reducing nursing workload
8	Ronquillo et al. (2021) [3]	United States	Delphi study	Primary study	International expert panel	AI decision support	Workforce analytics	Conceptual	Established international priorities for AI‐enabled nursing workforce management
9	Duffield et al. (2010) [23]	Australia	Observational study	Primary study	Hospitals	Predictive algorithmic modelling for skill‐mix optimisation	Skill‐mix optimisation	Implemented	Data‐driven staffing models improved workforce allocation and patient outcomes.
10	Girardon da Rosa et al. (2024) [24]	Brazil	Predictive modelling study	Primary study	Hospital	Artificial intelligence/machine learning–based predictive classifier model	Workload management	Evaluated	An AI‐based predictive classifier model was developed to assess nursing workload using electronic patient‐record data, demonstrating measurable predictive performance and the potential to automate workload assessment
11	Fazakarley et al. (2023) [25]	United Kingdom	Qualitative study	Primary study	NHS and UK healthcare/academic settings	AI in health care	Workforce workload, implementation readiness and organisational adoption	Implemented	Healthcare professionals generally perceived benefits from AI but identified organisational, regulatory, data‐governance and workload‐related barriers; concerns included data security, misdiagnosis and the potential for AI to increase staff workload
12	Renggli et al. (2025) [12]	Switzerland	Qualitative focus‐group study with preliminary AI implementation	Primary study	Hospital	AI‐based nurse scheduling using MIP, CP, GP and RL approaches	Nurse scheduling, workforce allocation, fairness and work–life balance	Pilot	Nurses and managers identified fairness, participation, flexibility and autonomy as key scheduling requirements. Participants anticipated that AI could improve efficiency, fairness and reduce administrative workload, whereas concerns included reliability, data quality, system failure and loss of human oversight
13	Kotp et al. (2025) [26]	Egypt/Qatar	Descriptive cross‐sectional survey	Primary study	Hospital	Predictive analytics	Nursing leadership and workforce management	Pilot	Nurse leaders demonstrated positive readiness for AI integration. Perceived benefits included improved workforce planning, managerial decision‐making and strategic leadership
14	Barker, Griffiths and Dall’Ora (2025) [27]	United Kingdom	Qualitative study	Primary study	Hospital network	Not applicable (contextual evidence for AI‐supported scheduling)	Shift scheduling	Conceptual	The study identified competing priorities influencing nurse shift scheduling, including fairness, flexibility, continuity of care, staff preferences and work–life balance. Findings suggest that future AI‐supported scheduling systems should incorporate these human and organisational factors to improve acceptability and implementation

*Note:* AI classification: During the two‐reviewer consensus process (Section 3.2), studies were carefully evaluated against the operational definition of AI. Duffield et al. (2010) were retained because it utilised predictive algorithmic modelling to optimise staffing ratios beyond conventional manual calculations. Girardon da Rosa et al. [24] were included because it developed and evaluated a machine learning–based predictive classifier using electronic patient‐record data to assess nursing workload, thereby demonstrating an identifiable AI component beyond conventional rule‐based digital technologies. The study was classified as evaluated because the predictive model was empirically assessed using quantitative performance metrics. Barker et al. [27] was retained as contextual primary evidence because, although it did not evaluate a specific AI system, it provided qualitative evidence on competing priorities in nurse scheduling that should inform the design and implementation of future AI‐supported scheduling systems.

**TABLE 3:** Characteristics of secondary studies on artificial intelligence in nursing workforce management.

No.	Author (year)	Country	Study design	Evidence type	Healthcare setting	AI technology	Workforce management domain	AI maturity level	Key findings
1	El Arab et al. (2025) [28]	Lebanon	Integrative review	Secondary study	Not applicable	Artificial intelligence, machine learning	Nursing workforce management	Not applicable (N/A)	Synthesised evidence on AI applications for workforce planning, staffing optimisation and managerial decision‐making
2	Hassanein et al. (2025) [8]	Egypt	Integrative review	Secondary study	Not applicable	Artificial intelligence, predictive analytics	Nursing management	Not applicable (N/A)	Reported opportunities for AI to improve operational efficiency while highlighting ethical and governance challenges
3	Kiwanuka et al. (2025) [9]	Finland/Canada	Scoping review	Secondary study	Not applicable	Artificial intelligence, workforce analytics	Nursing leadership and workforce management	Not applicable (N/A)	Mapped AI applications and identified major evidence gaps in implementation research
4	Ng et al. (2022) [7]	Singapore	Scoping review	Secondary study	Health care/clinical nursing care	Artificial intelligence	Nursing practice, clinical decision support and nursing management implications	Not applicable (N/A)	AI was applied to documentation, nursing diagnoses, care planning, patient monitoring, outcome prediction and wound management. The review highlighted AI’s potential to improve nursing care and emphasised the need for stronger real‐world evidence and AI education to prepare nurses for technological transformation
5	Bakken and Dreisbach (2022) [11]	United States	Perspective/state‐of‐the‐science analysis	Secondary study	Not applicable	Nursing informatics and data science	Workforce optimisation and informatics competency	Not applicable (N/A)	Highlighted the need for nursing data, informatics infrastructure and data‐science competencies to support future nursing decision‐making, system transformation and scalable nurse‐led interventions
6	Fagerström et al. (2017) [13]	Sweden	Integrative review	Secondary study	Not applicable	Clinical decision‐support systems	Workflow management	Not applicable (N/A)	Reviewed digital decision‐support technologies supporting nursing workflow efficiency
7	Booth et al. (2021) [29]	United Kingdom	Policy analysis	Secondary study	National healthcare system	Digital health and AI	Digital workforce readiness	Not applicable (N/A)	Highlighted organisational readiness and AI capability development for the nursing workforce
8	Morley et al. (2020) [30]	United Kingdom	Mapping review	Secondary study	Not applicable	Ethical AI	AI governance	Not applicable (N/A)	Proposed ethical principles including transparency, fairness, accountability and explainability
9	Yakusheva et al. (2025) [5]	United States	Analytical review/discussion paper	Secondary study	Not applicable	Artificial intelligence and AI‐enabled automation	Nursing workforce optimisation and staffing implications	Not applicable (N/A)	AI may reduce routine and administrative work, improve workforce efficiency and create more time for direct patient care and professional development; however, concerns regarding job displacement and the need for ethical, nurse‐led AI integration remain
10	Topaz and Pruinelli (2017) [31]	United States	Narrative review	Secondary study	Not applicable	Big data and machine learning	Nursing workforce planning	Not applicable (N/A)	Highlighted AI and predictive analytics as emerging tools for workforce planning
11	Shepherd and McCarthy (2025) [32]	United States	Narrative review	Secondary study	Hospital settings	Artificial intelligence	Nursing leadership and workforce management	Not applicable (N/A)	Synthesised current evidence on AI applications in nursing practice and management, highlighting AI’s potential to support workforce optimisation, managerial decision‐making, leadership development, education and ethical governance while emphasising the importance of responsible implementation and human oversight
12	Buchan and Catton (2020) [1]	International	Policy report	Secondary study	Global health systems	Workforce intelligence	Strategic workforce planning	Not applicable (N/A)	Advocated technology‐enabled workforce planning to address the global nursing shortage
13	Abdalkareem et al. (2023) [33]	Jordan	Scoping review	Secondary study	Not applicable	Artificial intelligence, machine learning, robotics	Nursing management and workforce applications	Not applicable (N/A)	Mapped AI applications across nursing domains including workforce scheduling and management. Highlighted major challenges including ethical concerns, need for proper training, infrastructure requirements and the necessity of involving nurses in AI development
14	Buchanan et al. (2020) [34]	Zealand/International	Scoping review	Secondary study	Not applicable	Artificial intelligence (general applications)	Workforce readiness and implementation acceptance	Not applicable (N/A)	Identified that nurses generally held positive attitudes toward AI but expressed concerns about job displacement, lack of knowledge and ethical issues. Emphasised that nursing leadership must address education and change management for successful AI integration

*Note:* AI maturity level is classified as ‘not applicable (N/A)’ for secondary study sources (e.g., reviews, policy reports), as these studies synthesise existing literature rather than reporting on the direct implementation or evaluation of a specific AI system.

### 4.3. AI Technologies Applied in Nursing Workforce Management

Several AI technologies were identified across the included studies.

*Among the 14 primary studies,* machine learning algorithms were the most frequently *applied technology,* particularly for workforce demand forecasting and staffing prediction [18, 23, 26]. Optimisation algorithms and intelligent scheduling systems were commonly applied to nurse rostering, shift allocation and preference‐based scheduling [12, 17, 20, 27]. Predictive analytics was primarily used to estimate staffing requirements, anticipate workload fluctuations and support proactive workforce planning [18, 23, 26, 31]. Decision‐support systems integrated organisational data to assist nurse managers in operational decision‐making [3, 32].

Several recent studies also explored human‐centred AI design approaches, explainable AI and AI‐assisted workforce analytics to improve transparency, fairness and user acceptance [6, 12].

### 4.4. Application Domains

Six major workforce management domains emerged from the evidence synthesis.

#### 4.4.1. Workforce Planning and Demand Forecasting

Numerous studies explored AI‐assisted workforce forecasting, enabling prediction of staffing requirements, patient demand and workforce capacity using routinely collected organisational data [18, 23, 26, 31].

#### 4.4.2. Nurse Scheduling and Rostering

Nurse scheduling represented one of the most frequently investigated application areas among *the primary studies*. AI‐supported scheduling systems aimed to improve roster fairness, accommodate staff preferences, optimise work–life balance and increase scheduling transparency [12, 17, 20, 27, 28].

#### 4.4.3. Workload Management

Machine learning models were used to assess and predict nursing workload using patient and nursing care data, supporting data‐driven workload assessment and workforce allocation [18, 23, 24].

#### 4.4.4. Workforce Sustainability and Burnout Prevention

Several publications highlighted the potential of predictive analytics to identify workforce stress, burnout risk, absenteeism and retention challenges before adverse outcomes occurred [6, 19, 28].

#### 4.4.5. Workforce Analytics

AI‐enabled workforce analytics supported organisational performance monitoring, human resource planning and strategic workforce decision‐making [3, 11, 22].

#### 4.4.6. Managerial Decision Support

Decision‐support systems were increasingly recognised as tools to assist nurse managers in evidence‐informed operational and strategic decision‐making by integrating multiple organisational data sources [6, 9, 26, 32].

### 4.5. Implementation Challenges

Analysis of the 14 primary studies identified several recurring implementation challenges at the organisational and technological levels. Ethical concerns, including transparency and algorithmic fairness, were reported in primary qualitative and implementation studies [3, 6, 22, 27, 30]. Technical challenges such as data quality and interoperability were highlighted across primary observational, predictive modelling and implementation studies [12, 24, 26]. Organisational barriers, including insufficient digital readiness and inadequate training, were also identified in the primary evidence [6, 19, 21, 25]. Furthermore, the secondary evidence sources (e.g., reviews and policy analyses) consistently contextualised these primary findings, emphasising that successful implementation broadly depends on active nurse involvement and robust governance [11, 13, 34].

### 4.6. Evidence Maturity

Although AI applications were identified across multiple workforce management domains, the overall primary evidence base remains at an early stage of maturity. Among the 14 primary studies, the majority were classified as being at early stages of AI maturity: 5 (35.7%) were categorised as conceptual and 3 (21.4%) as pilot. Four studies (28.6%) reached the implemented stage, whereas only two (14.3%) were classified as evaluated.

To operationalise the proposed conceptual framework, a cross‐tabulation of AI maturity levels across the six workforce management domains is presented in Table 1.

As illustrated in the heat map, AI‐supported nurse scheduling and managerial decision support received the most empirical attention; however, this evidence was primarily distributed across conceptual, pilot and implemented stages. Notably, rigorous methodological evaluation (evaluated stage) was concentrated exclusively in workload management. Strategic areas such as workforce sustainability and burnout prevention lacked any primary studies. Consequently, current evidence primarily demonstrates the potential feasibility of AI‐supported nursing workforce management rather than definitive evidence of effectiveness.

## 5. Discussion

### 5.1. Synthesis of Findings and Conceptual Framework

A major contribution of this review is the development of a conceptual framework integrating three complementary dimensions: AI technology, workforce management domain and AI maturity level (conceptual, pilot, implemented and evaluated). The operational definitions distinguishing these four maturity stages are detailed in Section 3.5 (Data Charting) to ensure transparency and replicability. Unlike previous reviews that classified studies primarily by AI methodology or clinical application [7–9], this framework provides a more comprehensive understanding of AI implementation within nursing management. It also explains why reported outcomes vary considerably across studies, as differences often reflect implementation maturity rather than inconsistencies in AI performance.

### 5.2. Differentiation From Existing Reviews

Although several recent reviews have examined AI in nursing, this review provides distinct contributions compared to overlapping literature. Kiwanuka et al. [9] specifically focused on *nurse leadership* and AI integration, whereas the current review encompasses a broader, operational spectrum of workforce management domains (e.g., scheduling, workload prediction, workforce analytics). Similarly, El Arab et al. [28] and Hassanein et al. [8] provided valuable integrative overviews of AI across clinical and operational nursing, but they did not classify evidence by implementation maturity. The primary added value of this review lies in its novel conceptual framework integrating AI technology, workforce domains and AI maturity levels. Unlike previous reviews that treated the AI literature as a homogeneous body of evidence, our framework is operationalised through explicit definitions, and the cross‐tabulation matrix (Table 1) enables researchers and nurse managers to visually distinguish between theoretical AI concepts and rigorously evaluated implementations, precisely identifying where empirical gaps exist.

To ensure clarity, the synthesis of findings is structured below around the review’s five research questions:

 
*RQ1: Which AI technologies have been applied?* Synthesis of the secondary evidence sources (reviews and policy reports) indicates that AI applications in the broader healthcare context have evolved beyond traditional nurse rostering systems toward more sophisticated technologies, including machine learning, predictive analytics, optimisation algorithms and decision‐support systems. This evolution reflects the broader digital transformation of health care and the shift from reactive staffing approaches to predictive, data‐driven management [4, 5, 30–32]. Furthermore, although generative AI has recently emerged as a promising tool for administrative support, documentation, education and managerial communication, evidence regarding its effectiveness in workforce management remains largely exploratory [8, 11, 13].
 
*RQ2: Which workforce management domains have been addressed?* Six principal domains were identified. AI‐supported nurse scheduling represents the most mature application area; this reflects the highly structured nature of scheduling tasks and the availability of measurable operational outcomes [12, 17, 20, 27]. Predictive workforce analytics has also advanced considerably. In contrast, AI applications supporting strategic nursing leadership, workforce sustainability, retention planning and organisational policy remain comparatively underdeveloped [9, 27].
 Notably, no included studies specifically examined the use of AI for nursing staff recruitment. Furthermore, although retention was identified as a relevant concept, it was not addressed as a standalone AI application; rather, it was exclusively discussed within the broader context of workforce sustainability and burnout prevention. The complete absence of AI applications dedicated to recruitment and standalone retention strategies represents a critical evidence gap in the current literature.
 
*RQ3: What organisational and workforce outcomes have been reported?* Reported outcomes varied significantly depending on implementation maturity. Early‐stage studies primarily highlighted anticipated benefits, such as improved scheduling transparency and data‐informed decision‐making. However, as demonstrated by the maturity heat map (Table 1), rigorous evaluations of actual organisational outcomes (e.g., long‐term efficiency, patient safety impacts) remain scarce. Consequently, the current evidence demonstrates promising potential rather than definitive effectiveness.
 
*RQ4: What implementation challenges have been identified? Among the primary studies, ethical concerns and technical barriers were the most frequently reported challenges. Importantly, when these primary findings are viewed alongside the secondary evidence, a clear sociotechnical perspective on digital transformation emerges, emphasising that* successful AI implementation depends not only on technological capability but also on organisational readiness, leadership commitment and workforce engagement [6, 21, 25, 30, 33]. Consequently, for nursing managers, AI must be viewed as a complementary decision‐support resource rather than a substitute for professional judgement. Successful implementation therefore requires transparent governance, explainable AI systems, continuous evaluation and active involvement of nurse managers throughout system design and implementation [,22,34].
 
*RQ5: What evidence gaps and future research priorities emerge?* The most critical gap identified through the maturity framework is the paucity of *evaluated* studies across all domains. Furthermore, the review highlights a marked geographical imbalance, with most empirical evidence originating from high‐income countries. Evidence from low‐ and middle‐income countries remains scarce, despite these countries experiencing some of the most severe nursing workforce shortages [1]. Future research must therefore shift from conceptual and pilot designs to multicentre, longitudinal implementation studies, particularly in diverse and underresourced healthcare settings, and regarding strategic leadership applications.

## 6. Implementation Challenges, Strengths, Limitations and Future Research

Integrating the primary empirical findings with the contextual insights from the secondary literature, several overarching challenges emerge. For instance, although primary studies highlighted trust and transparency at the user level [4, 6, 12], the secondary evidence sources broadened this perspective by emphasising macrolevel ethical governance [30]. Nurse managers were more likely to accept AI‐assisted recommendations when algorithms were explainable, incorporated staff preferences and allowed managerial oversight [4, 6, 12]. Conversely, opaque black‐box systems raised concerns regarding fairness, accountability and professional autonomy [22, 30].

Data quality and digital infrastructure also emerged as critical prerequisites. Predictive AI systems require accurate, standardised and interoperable workforce data; however, fragmented information systems and inconsistent documentation remain common barriers [11, 13, 24, 25]. Consequently, investments in data governance, interoperability and digital infrastructure should accompany AI implementation initiatives.

Ethical governance represents another essential requirement. AI‐supported workforce decisions must be implemented within frameworks that emphasise transparency, fairness, accountability, privacy and human oversight to minimise algorithmic bias and protect workforce well‐being [30]. Similarly, AI literacy and digital competencies among nurse managers should be strengthened through leadership development and continuing professional education [11, 19, 29, 33].

This review also highlights important methodological limitations within the existing evidence. Most empirical studies employed observational, qualitative or single‐centre designs, whereas rigorous multicentre implementation studies, longitudinal evaluations and economic analyses remain limited [9]. Consequently, current evidence provides stronger support for implementation feasibility than for long‐term organisational effectiveness.

Future research should prioritise multicentre implementation studies evaluating AI across diverse healthcare systems, particularly in low‐ and middle‐income countries. Greater attention should also be directed toward strategic workforce applications, including retention prediction, burnout prevention, competency‐based workforce planning, succession planning and leadership decision support. Furthermore, future investigations should adopt human‐centred AI approaches that evaluate not only algorithm performance but also organisational trust, workforce engagement, equity, leadership effectiveness and patient outcomes [12, 26, 34].

This review has several strengths, including adherence to PRISMA‐ScR guidance [14, 16], comprehensive database searching, inclusion of multiple evidence types and development of a novel conceptual framework integrating AI technology, workforce management domains and implementation maturity. However, no formal methodological quality appraisal was undertaken, only English‐language publications were included, and substantial heterogeneity across studies precluded quantitative synthesis. Furthermore, the exclusion of grey literature (e.g., conference abstracts and dissertations) represents a notable limitation. Given that early‐stage AI pilot implementations in health systems are frequently reported in conference proceedings before peer‐reviewed journals, this exclusion may bias the review’s evidence base toward more mature or conceptual academic publications, potentially underrepresenting frontline pilot activities. These limitations should be considered when interpreting the findings.

Furthermore, regarding emerging technologies, the search strategy intentionally excluded proprietary terms (e.g., ‘ChatGPT’, ‘Large Language Models’) to avoid nonempirical literature. Consequently, the review may underidentify the most recent generative AI literature specifically applied to nursing workforce management, representing a limitation in capturing this rapidly evolving subfield.

## 7. Implications for Nursing Management

The findings have important implications for nursing management. AI offers promising opportunities to support evidence‐informed workforce planning, staffing optimisation and operational decision‐making, but successful implementation requires more than technological innovation. Organisations should prioritise organisational readiness through investment in digital infrastructure, workforce education, governance frameworks and high‐quality workforce data [21, 25, 26]. Equally important is the active involvement of nurse managers in AI design, implementation and evaluation to ensure alignment with professional nursing values and patient‐centred care [19, 32].

Overall, AI should be considered an enabling technology that strengthens rather than replaces human leadership in nursing workforce management [6, 32]. Although current evidence suggests considerable promise, robust implementation studies evaluating long‐term organisational outcomes remain limited. Future progress will depend on responsible governance, interdisciplinary collaboration, organisational readiness and rigorous evaluation of real‐world implementation. The conceptual framework proposed in this review provides a structured foundation for guiding future research and supporting the sustainable integration of AI into nursing workforce management.

## 8. Conclusion

This scoping review highlights that although AI has considerable potential to transform nursing workforce management, the current evidence base remains predominantly at the conceptual or pilot stage. The novel conceptual framework introduced in this review, integrating AI technologies, workforce domains and implementation maturity, provides a structured tool for researchers and managers to identify precisely where empirical evidence is lacking. Future progress requires a decisive shift from theoretical models to rigorous, multicentre implementation studies, particularly in low‐ and middle‐income settings, ensuring that AI is integrated as a complement to, rather than a replacement for, human nursing leadership.

## 9. Key Messages

• AI in nursing workforce management shows high potential but currently lacks rigorous, large‐scale evaluation evidence.
• A novel maturity‐based framework reveals that strategic applications (e.g., retention, sustainability) significantly lag behind operational areas (e.g., scheduling).
• Successful AI integration relies on sociotechnical factors (governance, readiness, user engagement) as much as on algorithmic capability.
• Future research must prioritise longitudinal, real‐world evaluations and human‐centred AI design in diverse healthcare settings.

## Author Contributions

Study design: Rafat Rezapour‐Nasrabad and Sina Nasrollahi‐Nasrabad. Data collection: Rafat Rezapour‐Nasrabad. Data analysis: Rafat Rezapour‐Nasrabad and Sina Nasrollahi‐Nasrabad. Study supervision: Sina Nasrollahi‐Nasrabad. Manuscript writing: Rafat Rezapour‐Nasrabad. Critical revisions for important intellectual content: Sina Nasrollahi‐Nasrabad.

## Funding

No funding was obtained for this research.

## Ethics Statement

Ethical approval was not required for this study because it is a scoping review based exclusively on publicly available published literature and did not involve human participants, identifiable personal data or primary data collection.

## Conflicts of Interest

The authors declare no conflicts of interest.

## Supporting Information

Additional supporting information can be found online in the Supporting Information section.

## Supporting information


**Supporting Information** The following supporting information is provided to enhance the transparency and reproducibility of this scoping review’s methodology: Supporting File 1: The complete electronic database search strategies executed in PubMed/MEDLINE, Scopus, Web of Science Core Collection, CINAHL Complete and IEEE Xplore. This file details the controlled vocabulary, free‐text keywords, Boolean operators, search limits applied and the total yield for each database, including the supporting top‐up search. Supporting File 2: The completed Preferred Reporting Items for Systematic Reviews and Meta‐Analyses Extension for Scoping Reviews (PRISMA‐ScR) checklist, mapping each reporting item to the corresponding page number in the main manuscript. Supporting File 3: The a priori review protocol developed before the commencement of the study. It outlines the review objectives, the Population–Concept–Context (PCC) eligibility criteria, information sources, the data charting plan and the planned synthesis approach to ensure full methodological transparency.

## Data Availability

All data used in this scoping review were extracted from publicly available published literature. The extracted study characteristics, data‐charting framework and database‐specific search strategies are provided in the supporting information accompanying this article.
