# Supplementary material for: Digital Innovations in Nursing Workforce Management: A Scoping Review of Advanced Applications and Implementation Challenges
Source: J Nurs Manag. 2026 Sep 14;2026:1858294. doi: 10.1155/jonm/1858294 (PMC13575483; doi:10.1155/jonm/1858294)
Supplement: Supplementary file 1 [file JONM-2026-1858294-s001.zip › Supplementary File 3 - A Priori Review Protocol.docx]

**Review Protocol: Digital Innovations in Nursing Workforce Management**

**Review Objectives:** To systematically map the existing literature on AI applications in nursing workforce management, synthesise current evidence across major workforce management domains, identify reported organisational outcomes and implementation challenges, and highlight evidence gaps.

**Eligibility Criteria (PCC Framework):**

- **Population:** Registered nurses, nurse managers, nursing teams, or healthcare organisations where nursing workforce management is the primary focus.
- **Concept:** Artificial intelligence (machine learning, predictive analytics, optimization algorithms, NLP, intelligent decision-support) applied to nursing workforce management activities (planning, staffing, scheduling, workload, recruitment, retention, analytics). Excludes conventional digital technologies without explicit AI functionality.
- **Context:** Hospitals, community healthcare organizations, long-term care facilities, academic health centres.
- **Types of Sources:** Empirical quantitative, qualitative, mixed-methods, implementation, modelling, simulation studies, and evidence syntheses. Excludes editorials, opinion papers, conference abstracts, and protocols.

**Information Sources:** PubMed/MEDLINE, Scopus, Web of Science Core Collection, CINAHL Complete, and IEEE Xplore (from inception to May 2025). Reference lists of included studies will also be screened.

**Search Strategy:** Controlled vocabulary (e.g., MeSH) combined with free-text keywords using Boolean operators (AND, OR) combining three concepts: (1) Artificial Intelligence, (2) Nursing, and (3) Workforce Management.

**Data Charting Plan:** Data will be extracted independently by two reviewers using a standardized form including: Author(s), Year, Country, Study design, Evidence type, Healthcare setting, AI technology, Workforce management domain, AI Maturity Level, and Key Findings. Discrepancies will be resolved by consensus.

**Synthesis:** Descriptive numerical analysis and thematic synthesis. No meta-analysis will be undertaken.

---------------------------------------------------------------------------------------------------------------------

***Note****: Following the initial protocol, a top-up search was conducted from June 2025 to August 2026, yielding no new studies.*
