# Supplementary material for: Digital Innovations in Nursing Workforce Management: A Scoping Review of Advanced Applications and Implementation Challenges
Source: J Nurs Manag. 2026 Sep 14;2026:1858294. doi: 10.1155/jonm/1858294 (PMC13575483; doi:10.1155/jonm/1858294)
Supplement: Supplementary file 1 [file JONM-2026-1858294-s001.zip › Supplimentary file 1.docx]

**Supplementary File 1: Complete Electronic Database Search Strategies**

Searches were conducted from database inception to 31 May 2025. Limits applied to all databases: English language, peer-reviewed journals. The search strategies were developed in collaboration with a research librarian.

**1. PubMed / MEDLINE (via PubMed)**

**Database Interface:** PubMed.gov

**Search Date:** 31 May 2025

**Total Results:** [158]

| Search Set | Search Query |
| --- | --- |
| #1 | "Artificial Intelligence"[MeSH] OR "Machine Learning"[MeSH] OR "Deep Learning"[MeSH] OR "Neural Networks, Artificial"[MeSH] OR "Decision Support Systems, Clinical"[MeSH] |
| #2 | "Artificial intelligence"[tiab] OR "machine learning"[tiab] OR "deep learning"[tiab] OR "predictive analy*"[tiab] OR "decision support*"[tiab] OR "algorithm*"[tiab] |
| #3 | #1 OR #2 |
| #4 | "Nursing"[MeSH] OR "Nurses"[MeSH] |
| #5 | nurs*[tiab] |
| #6 | #4 OR #5 |
| #7 | "Health Personnel"[MeSH] OR "Personnel Staffing and Scheduling"[MeSH] OR "Personnel Management"[MeSH] |
| #8 | "workforce"[tiab] OR "staffing"[tiab] OR "schedul*"[tiab] OR "roster*"[tiab] OR "human resource*"[tiab] OR "workload"[tiab] OR "nurse management"[tiab] |
| #9 | #7 OR #8 |
| #10 | #3 AND #6 AND #9 |
| #11 (Final) | #10 Filters: English |

**2. CINAHL Complete (via EBSCOhost)**

**Database Interface:** EBSCOhost

**Search Date:** 31 May 2025

**Total Results:** [89]

| Search Set | Search Query |
| --- | --- |
| S1 | (MH "Artificial Intelligence") OR (MH "Machine Learning") OR (MH "Deep Learning") OR (MH "Algorithms") OR (MH "Decision Support Systems") |
| S2 | TI (“artificial intelligence" OR "machine learning" OR "deep learning" OR "predictive analy*" OR "decision support*”) OR AB (“artificial intelligence" OR "machine learning" OR "deep learning" OR "predictive analy*" OR "decision support*”) |
| S3 | S1 OR S2 |
| S4 | (MH "Nursing+") OR (MH "Nurses+") |
| S5 | TI (nurs*) OR AB (nurs*) |
| S6 | S4 OR S5 |
| S7 | (MH "Personnel Staffing and Scheduling") OR (MH "Staffing") OR (MH "Workload") OR (MH "Personnel Management") |
| S8 | TI (workforce OR staff* OR schedule* OR roster* OR "human resource*" OR workload) OR AB (workforce OR staff* OR schedule* OR roster* OR "human resource*" OR workload) |
| S9 | S7 OR S8 |
| S10 (Final) | S3 AND S6 AND S9 *(Limiters: English Language, Peer Reviewed)* |

**3. Scopus**

**Database Interface:** Scopus

**Search Date:** 31 May 2025

**Total Results:** [215]

| Search Set | Search Query |
| --- | --- |
| Final Query | TITLE-ABS-KEY ( "artificial intelligence" OR "machine learning" OR "deep learning" OR "predictive analy*" OR "decision support*" OR algorithm* ) AND TITLE-ABS-KEY ( nurs* OR "nursing workforce" ) AND TITLE-ABS-KEY (workforce OR staff* OR schedul* OR roster* OR "human resource*" OR workload) AND ( LIMIT-TO ( LANGUAGE , "English" ) ) AND (LIMIT-TO ( SRCTYPE , "j" ) ) |

**4. Web of Science Core Collection**

**Database Interface:** Clarivate Web of Science

**Search Date:** 31 May 2025

**Total Results:** [124]

| Search Set | Search Query |
| --- | --- |
| Final Query | TS= (“artificial intelligence" OR "machine learning" OR "deep learning" OR "predictive analy*" OR "decision support*" OR algorithm*) AND TS= (nurs* OR "nursing workforce”) AND TS= (workforce OR staff* OR schedul* OR roster* OR "human resource*" OR workload) AND LANGUAGE: (English) AND DOCUMENTTYPES: (Article OR Review) |

**5. IEEE Xplore**

**Database Interface:** IEEE Xplore Digital Library

**Search Date:** 31 May 2025

**Total Results:** [34]

| Search Set | Search Query |
| --- | --- |
| Final Query | ("Abstract”: “artificial intelligence" OR "machine learning" OR "deep learning" OR "predictive analytics" OR "decision support") AND ("Abstract”: nurs* OR "Index Terms”: nurs*) AND ("Abstract”: “workforce" OR "Abstract”: staffing OR "Abstract": scheduling OR "Abstract": rostering OR "Abstract”: “human resource") *(Filter: English Language)* |

**Top-Up Search Update (1 June 2025 to 10 August 2026):** To ensure the evidence base reflects the latest developments in this rapidly evolving field, a supplementary top-up search was executed across the same five electronic databases (PubMed/MEDLINE, Scopus, Web of Science Core Collection, CINAHL Complete, and IEEE Xplore) on 10 August 2026.

**Search Parameters:**

- **Date Filter:** 1 June 2025 to 10 August 2026
- **Search Strategy:** Identical to the main search strategies detailed above.
- **Limits:** English language, peer-reviewed journals.

**Results:** No additional eligible studies were identified from this updated search. Therefore, the total number of included studies remains 28.
